# Real-world in-hospital outcomes and potential predictors of heart failure in primigravid women with heart disease in Southwestern China

**DOI:** 10.1186/s12884-020-03058-9

**Published:** 2020-06-23

**Authors:** Wuwan Wang, Lu Wang, Panpan Feng, Xiyao Liu, Rui Xiang, Li Wen, Wei Huang

**Affiliations:** 1 Department of Cardiology, The First Affiliated Hospital of Chongqing Medical University, Chongqing, China; 2 Department of Obstetrics, The First Affiliated Hospital of Chongqing Medical University, Chongqing, China

**Keywords:** Pregnancy, Cardiac disease, Pulmonary hypertension, Risk factors

## Abstract

**Background:**

Little is known about the status of maternal, obstetric, and neonatal complications and the potential predictors of developing heart failure (HF) in mothers with underlying heart disease (HD) in Southwestern China.

**Methods:**

The eligible records from the YiduCloud database from December 1, 2010 to December 31, 2019 were screened. The maternal clinical characteristics and the in-hospital outcomes were collected and compared in primigravid women with and without HD. The HD subtypes analyzed included valvular HD (VHD), cardiomyopathy, adult congenital HD (ACHD), pulmonary hypertension (PH), and other cardiac conditions.

**Results:**

Among 45,067 primigravid women, 508 (1.1%) had HD, in which 207 (41%) had ACHD, 66 (13%) had VHD, 84 (17%) had cardiomyopathy, 7 (1%) had PH, and 144 (28%) had other cardiac diseases. The maternal cardiac events and the neonatal complications occurred in 28% and 23.3%, respectively, of women with HD and were predominant in the PH group. In multivariable regression, HF was associated with the New York Heart Association (NYHA) class ≥3 (OR = 15.9, 95% confidence interval [CI] = 2.5–99.7; *P* = 0.003), heart rate ≥ 100 bpm (OR = 3.8, 95% CI = 1.1–13.5; *P* = 0.036), ejection fraction ≤60% (OR = 6.4, 95% CI = 2.0–21.0; *P* = 0.002) and left ventricular end-diastolic diameter ≥ 50 mm (OR = 3.4, 95% CI = 1.1–11.2; *P* = 0.041) at the beginning of pregnancy.

**Conclusions:**

Maternal and neonatal complications are higher in primigravid women with HD particularly in the PH group compared with primigravid women without HD. Women with HD should be guided on the potential predictors for HF and closely monitored during pregnancy to reduce maternal and neonatal complications.

## Background

Heart disease (HD) is the leading cause of nonobstetric maternal mortality in China and other countries [1, 2]. HD in pregnant women represents a spectrum of pathology that includes cardiomyopathies, valvular HD (VHD), adult congenital HD (ACHD), and pulmonary hypertension (PH) and has not been clearly defined [3]. Recent epidemiological data suggest that the prevalence of maternal HD during pregnancy is rising [3, 4]. Moreover, some other cardiac conditions (including primary arrythmia, coronary HD, anemic HD, and hyperthyroid HD) are gradually increasing. Cardiac output increases by 30–50% during pregnancy. Increased stroke volume and heart rate are also observed in this special status [5]. Women with underlying HD often have high risk for various complications due to the challenge of the adaptation toward increasing circulating blood volume. Heart failure (HF), a complex clinical syndrome induced by increasing cardiac workload, is considerably associated with neonatal and fetal clinical events [6–8]. A prior study from the prospective worldwide Registry of Pregnancy and Cardiac Disease has identified the independent predictive risk factors for cardiac complications as the baseline New York Heart Association (NYHA) functional class higher than II, WHO category ≥3, signs of HF, cardiomyopathy, or PH [9]. No prior study has identified the in-hospital complications and the potential predictors of HF in mothers with HD in Southwestern China. In this study, we sought to evaluate the prevalence of having HD and the maternal, obstetric, and neonatal outcomes in pregnant women with or without multiple forms of HD from the YiduCloud database in a contemporary era in Southwestern China. Furthermore, we identified the potential predictors of developing HF in pregnant women with HD.

## Methods

### Data source and study population

**Fig. 1:**
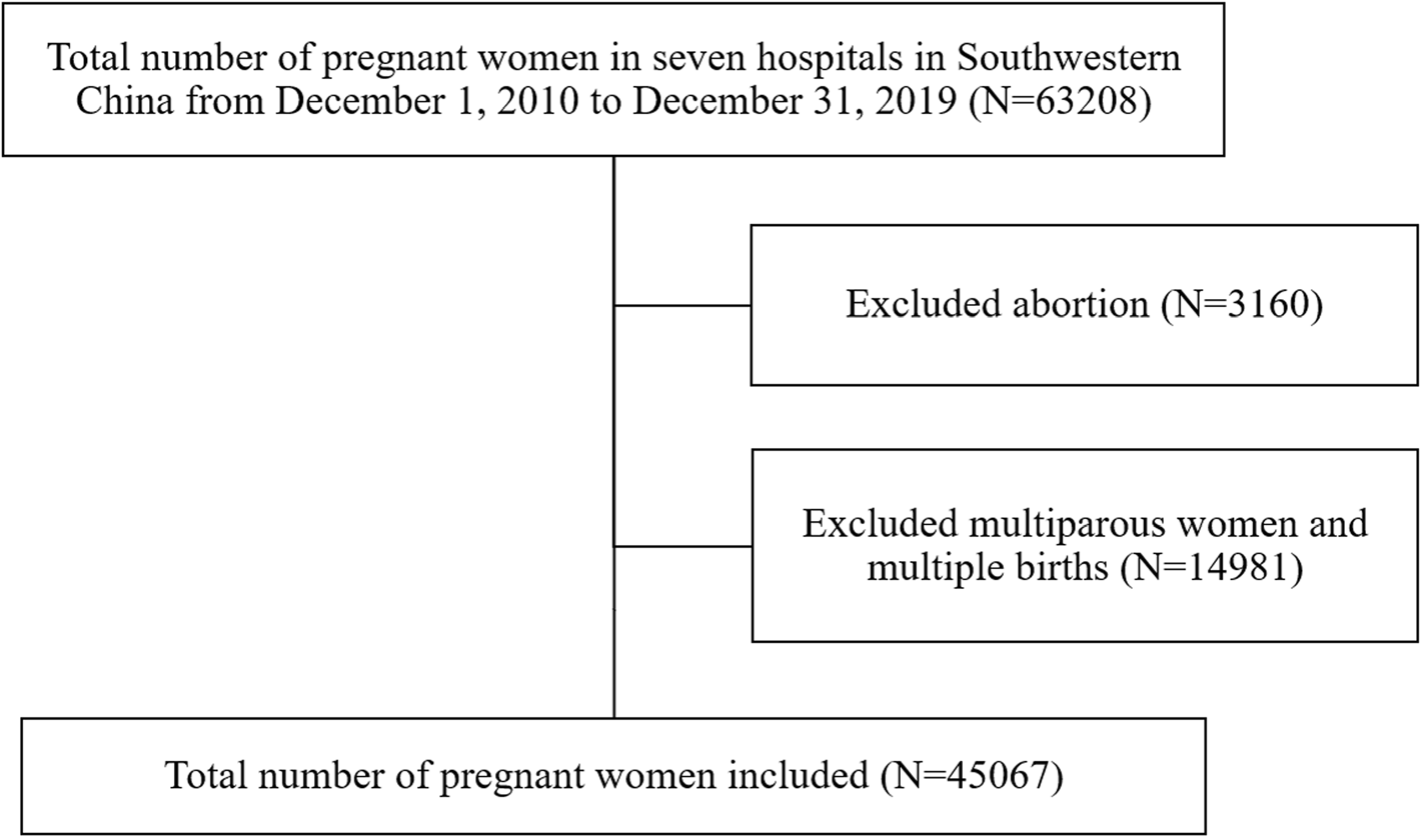
Flow chart of the study population

The YiduCloud database, which contained the medical records of 12,025,465 patients from seven main medical centers in Southwestern China, was used to characterize outcomes from December 1, 2010 to December 31, 2019 (Fig. 1). All primigravid women (excluding abortion, multiparous women, and multiple births) were eligible for inclusion in the present analysis. The cardiac diseases of a pregnant woman in the analysis included VHD, cardiomyopathy, ACHD, PH, and other cardiac conditions (primary arrythmia, coronary HD, anemic HD, and hyperthyroid HD). Only primigravid women with available data and unique medical identified numbers during the study period were included in the analysis. International Classification of Diseases, Tenth Revision, Clinical Modification (ICD-10-CM) codes were used in the diagnosis of the patients from the seven hospitals to identify pregnancy, forms of HD, and outcomes (Table S1). Patients aged < 18 years and with missing information (including unique patient identifiers) were excluded. Records with the same date of birth, admission date, discharge date, and facility name were considered as pure duplications, and only one of such records was kept. The data set was analyzed retrospectively, and the informed consent requirement was waived.

This study was a retrospective study that did not involve interactions with the study subjects. The data were from the Yiducloud (Chongqing) Technology Co., Ltd. and the cooperative Medical Data Science Academy of Chongqing Medical University, which accumulated and verified the patients’ chart reviews from seven main medical centers in Southwestern China. The authors were approved direct data access on the platform system with unique codes. All the information on the platform was anonymous and had unique identified codes for privacy protection. The data were not open access.

### Baseline characteristics

The clinical and the demographic characteristics of patients with and without HD were collected first. In the patients’ characteristics, the hypertensive disorders of pregnancy were defined and classified into (a) hypertension known before pregnancy or present in the first 20 weeks and (b) hypertension arising de novo at or after 20 weeks on the basis of the International Society for the Study of Hypertension in Pregnancy Classification, Diagnosis, and Management Recommendations for International Practice [10]. For patients with and without HF, the baseline characteristic data were obtained using the records at the first prenatal visit or the first pregnancy visit. The baseline data included age, etiologic classification, smoking, drinking, body mass index (BMI), diabetes mellitus, NYHA classification, blood pressure, echocardiographic parameters, blood lipid, B-type natriuretic peptide (BNP), and cardiac medication use. However, hypertensive disorders and total charge were obtained from the discharge records of each patient, which were not presented before pregnancy or at the first prenatal visit. The hypertensive disorder of pregnancy was included as a factor to assess the influence on HF in logistic regression. Women with hypertensive disease of pregnancy are more likely to be readmitted with HF [11].

### Outcome measures

The information for the present analysis was obtained through the review of the institutional database. The data had been entered on the discharge of each patient. Primary outcomes of interest were major adverse cardiac events (MACEs), obstetric complications and neonatal adverse clinical events (NACEs). Secondary outcomes included non-MACE and non-NACE outcomes. MACEs were defined as a composite of arrhythmia, shock, cerebrovascular events, HF, respiratory failure, in-hospital death, pulmonary embolism, and the dissection of any artery [3, 12, 13]. The cardiac procedural intervention was also recorded for patients with and without HD. The non-MACE maternal outcomes included acute renal failure and total hospital charges. The obstetric complications included adherent placenta, placental abruption, breech delivery, disseminated intravascular coagulation, early or threatened labor, known or suspected fetal abnormality, vaginal laceration, long labor, placental insufficiency, placenta previa, precipitate labor, premature rupture of membranes, polyhydramnios, postpartum hemorrhage, and infection. Neonatal adverse clinical events (NACEs) were a composite of fetal death (in utero), neonate death (within 30 days of birth), prematurity (< 37 weeks), intrauterine growth restriction, respiratory distress syndrome, and intracranial cerebral events. The non-NACE variables, including infants with low birth weight (weight < 2500 g) and fetal macrosomia (weight > 4000 g), were also measured. HF (during or after pregnancy) was defined in these patients with various underlying HDs as a clinical syndrome characterized by specific symptoms (e.g., dyspnea and fatigue) and signs of fluid retention (e.g., edema and rales). HF was judged by the treating cardiologist in accordance with the ACC/AHA guidelines [14]. The subsequent predictor selection for HF in univariable and multivariable logistic regression analyses was based on the baseline characteristic data.

### Statistical analysis

Results were presented as descriptive and inferential statistics. One-sample Kolmogorov–Smirnov tests and histograms were used to check the normality of continuous data. Normally distributed continuous data were presented as mean ± SD, whereas data that were not normally distributed were presented as median with IQR. The continuous variables (including age, BMI, total hospital charge, blood pressure, ejection fraction, fractional shortening, left ventricular end-diastolic diameter, low-density lipoprotein, total cholesterol, total triglyceride, and brain natriuretic peptide) were compared using one-way ANOVA or the Kruskal–Wallis test. The Levene statistics was performed initially to test the homogeneity of variances. If normalized distribution and homoscedasticity were being assessed, one-way ANOVA was used. Otherwise, the nonparametric Kruskal–Wallis test was used. The categorical variables were presented as percentage. The enumeration data were compared using the Pearson χ2 test with *P* values from the Monte Carlo simulation and the Fisher exact test when appropriate. The baseline characteristics of patients with and without HF were reported before the independent predictors of HF were analyzed in the logistic regression model. The univariable logistic regression analysis was conducted to identify the patient characteristics associated with HF. The multivariable adjusted regression model was used to examine the predictors of HF. And variables with an increased incidence of the studied endpoints (*P* <  0.15) in the univariable analysis or with a prior given clinical relevance entered the final multivariable regression analysis. *P* <  0.05 (two-sided test) was considered statistically significant. For both models, the odds ratio represented the comparison of risk between two groups. Statistical analysis was performed using the SPSS software (Version 24.0; SPSS Inc., Chicago, IL; RRID: SCR_002865).

## Results

**Fig. 2:**
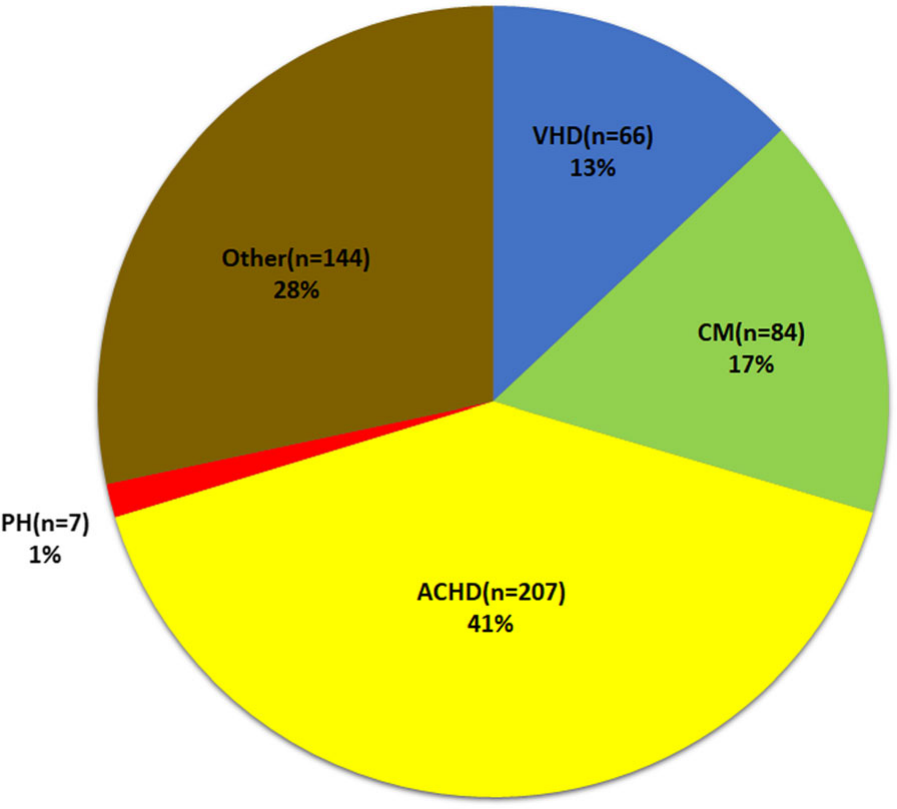
Prevalence of pregnancy and heart disease of various etiologies in Southwestern China, 2010–2019. ACHD, adult congenital heart disease; CM, cardiomyopathy; PH, pulmonary hypertension; VHD, valvular heart disease

A total of 45,067 primigravid women who met our criteria were identified. Of the 45,067 women, 508 (1.1%) had HD, whereas 44,559 (98.9%) did not have HD. Of the women with HD, 66 (13%) had VHD, 84 (17%) had cardiomyopathy, 207 (41%) had ACHD, 7 (1%) had PH, and 144 (28%) had other cardiac diseases (Fig. 2).

### Patient characteristics

The clinical and the demographic characteristics of all women with (*n* = 508) and without (*n* = 44,559) HD are summarized in Table 1. Compared with those without HD, the patients with HD had greater likelihood to be non-Han, self-supporting, and higher prevalence of hypertension (known before pregnancy or present in the first 20 weeks) and cesarean delivery (*P* <  0.05). However, diabetes mellitus and hypertension (arising de novo at or after 20 weeks) were more common in mothers without HD than in mothers with HD (*P* <  0.05).

**Table 1:** Patient Characteristics of Primigravid Women with and without HD in Southwestern China

	HD (*n* = 508)	No HD (*n* = 44,559)	P Value	VHD (*n* = 66)	CM (*n* = 84)	ACHD (*n* = 207)	PH (*n* = 7)	Other (*n* = 144)	*P* value
Age (years)	30.5 ± 5.3	31.1 ± 5.3	0.013^a^	32.0 (28.0, 37.0)	30.5 (27.0, 34.8)	30.0 (27.0, 33.0)	28.0 (26.0, 32.0)	29.0 (26.3, 32.0)	0.010^b^
Age group (years)									
18–25	81 (15.9)	6169 (13.8)	0.333	5 (7.6)	17 (20.2)	31 (15.0)	1 (14.3)	27 (18.8)	0.036
26–35	334 (65.7)	29,549 (66.3)		40 (60.6)	49 (58.3)	144 (69.6)	6 (85.7)	95 (66.0)	
> 35	93 (18.3)	8841 (19.8)		21 (31.8)	18 (21.4)	32 (15.5)	0 (0.0)	22 (26.4)	
Ethnic Han, *n*	499 (98.1)	44,203 (99.2)	0.018	65 (98.2)	84 (100.0)	201 (97.1)	7 (100.0)	142 (98.3)	0.674
Insurance status									
Self-supporting (%)	49.6	42.5	< 0.0001	50.0	47.6	55.1	28.6	43.8	0.004
Working (%)	11.6	19.2		4.5	4.8	9.7	14.3	21.5	
Residents (%)	18.5	18.3		19.7	20.2	17.9	42.9	16.7	
Other (%)	20.3	20.0		25.8	27.4	17.4	14.3	18.1	
Smoking (%)	1.8	0.7	0.094	0.0	2.3	3.3	0.0	0.0	0.464
Drinking (%)	1.4	0.5	0.107	0.0	2.3	1.6	0.0	1.6	0.908
BMI (kg/m^2^)	26.4 ± 3.9	26.7 ± 5.3	0.596^a^	26.2 ± 2.6	27.9 ± 8.0	25.9 ± 3.1	.	26.9 ± 2.8	0.368^b^
Obesity (%)	2.8	4.7	0.348	0.0	16.7	1.8	.	0.0	0.091
Hypertension known before pregnancy or present in the first 20 weeks	3 (0.6)	159 (0.4)	< 0.0001	1 (1.5)	0 (0.0)	2 (1.0)	0 (0.0)	0 (0.0)	0.582
Hypertension arising de novo at or after 20 weeks	29 (2.5)	1110 (5.7)	< 0.0001	4 (6.1)	10 (11.9)	9 (4.3)	1 (14.3)	5 (3.5)	0.061
Diabetes mellitus	40 (7.9)	6576 (14.8)	< 0.0001	5 (7.6)	8 (9.5)	14 (6.8)	0 (0.0)	13 (9.0)	0.820
Delivery type									
Cesarean delivery (%)	74.3	39.3	< 0.0001	78.8	88.2	74.1	83.3	64.7	0.119
Vaginal or assisted delivery (%)	25.7	60.7		21.2	11.8	25.9	16.7	35.3	

Data are shown as mean ± SD or median (IQR) for continuous outcomes and *n* (%) for categorical outcomes. *P* values were based on one-way ANOVA or the Kruskal–Wallis test for continuous outcomes and the Pearson chi-squared test or the Fisher exact test for categorical outcomes. The test utilization in continuous variables was presented with ^a^ for ANOVA and ^b^ for the Kruskal–Wallis test. ACHD, adult congenital heart disease; BMI, body mass index; CM, cardiomyopathy; HD, heart disease; PH, pulmonary hypertension; VHD, valvular heart disease

### Maternal cardiac outcomes

**Fig. 3:**
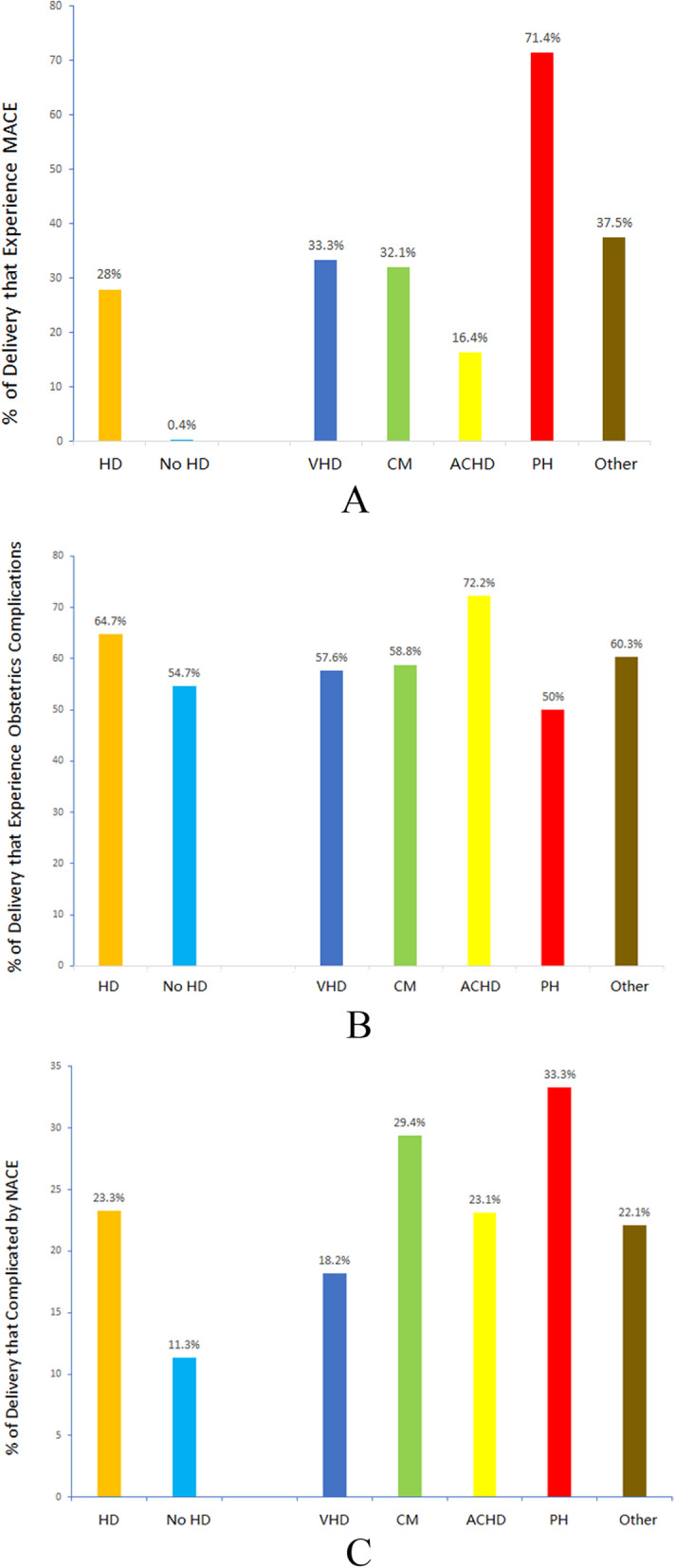
Maternal, obstetric, and neonatal adverse events in pregnant women with heart disease. **a** Maternal major adverse events in pregnant women with HD and by HD subtype. **b** Obstetric complications in pregnant women with HD and by HD subtype. **c** Neonatal complications in offspring of women with HD. ACHD, adult congenital heart disease; CM, cardiomyopathy; HD, heart disease; MACE, major adverse cardiac event; NACE, neonatal adverse clinical events; PH, pulmonary hypertension; VHD, valvular heart disease

The adverse maternal cardiac events and the HD subtypes are listed in Table 2 and Fig. 3a. Compared with those without HD, the patients with HD had higher rates of MACEs (28.0% vs. 0.4%; *P* <  0.0001), which was predominantly because of the higher rates of arrhythmia (14.6% vs. 0.1%) and HF (14.4% vs. 0.05%) (both *P* <  0.0001). The MACE rates were highest in women with PH and lowest in women with ACHD. The patients with PH had the highest rates of HF, whereas the patients in the other HD groups commonly had arrhythmia. As for the non-MACE outcomes, women with HD experienced higher total hospital charges (9581 ± 16,893 RMB vs. 5212 ± 3350 RMB; *P* <  0.0001) and rate of acute renal failure than women without HD (1.0% vs. 0.0%; *P* <  0.0001).

**Table 2:** Maternal Outcomes of Women with and without HD in Southwestern China

	HD (*n* = 508)	No HD (*n* = 44,559)	*P* value	VHD (*n* = 66)	CM (*n* = 84)	ACHD (*n* = 207)	PH (*n* = 7)	Other (*n* = 144)	*P* value
MACE	142 (28.0)	176 (0.4)	< 0.0001	22 (33.3)	27 (32.1)	34 (16.4)	5 (71.4)	54 (37.5)	< 0.0001
Arrythmia	74 (14.6)	51 (0.1)	< 0.0001	5 (7.6)	10 (11.9)	10 (4.8)	2 (18.6)	47 (32.6)	< 0.0001
Shock	5 (1.0)	98 (0.2)	0.002	0 (0.0)	3 (3.6)	0 (0.0)	0 (0.0)	2 (1.4)	0.057
Cerebral events	1 (0.2)	1 (0.0)	0.022	0 (0.0)	0 (0.0)	1 (0.5)	0 (0.0)	0 (0.0)	1.000
Heart failure	73 (14.4)	24 (0.05)	< 0.0001	17 (25.8)	18 (21.4)	28 (13.5)	3 (42.9)	7 (4.9)	< 0.0001
Respiratory failure	3 (0.6)	21 (0.0)	< 0.0001	1 (1.5)	0 (0.0)	2 (1.0)	0 (0.0)	0 (0.0)	0.405
In-hospital death	2 (0.4)	0 (0.0)	< 0.0001	0 (0.0)	2 (2.4)	0 (0.0)	0 (0.0)	0 (0.0)	0.071
Pulmonary embolism	1 (0.2)	2 (0.0)	0.03	0 (0.0)	1 (1.2)	0 (0.0)	0 (0.0)	0 (0.0)	0.309
Dissection of any artery	0 (0.0)	0 (0.0)	.	0 (0.0)	0 (0.0)	0 (0.0)	0 (0.0)	0 (0.0)	.
Cardiac procedural intervention	2 (0.4)	1 (0.0)	< 0.0001	0 (0.0)	0 (0.0)	1 (0.5)	0 (0.0)	1 (0.7)	1.000
Non-MACE outcomes									
Acute renal failure	5 (1.0)	10 (0.0)	< 0.0001	2 (3.0)	1 (1.2)	2 (1.0)	0 (0.0)	0 (0.0)	0.222
Total hospital charge (RMB)	9581 ± 16,893	5212 ± 3350	< 0.0001^a^	5402 (2605, 9936)	9158 (5363, 13,315)	6227 (4398, 9562)	20,102.	5578 (3482, 8283)	0.026^a^

Data are shown as mean ± SD or median (IQR) for continuous outcomes and *n* (%) for categorical outcomes. P values were based on one-way ANOVA or the Kruskal–Wallis test for continuous outcomes and the Pearson chi-squared test or the Fisher exact test for categorical outcomes. The test utilization in continuous variables was presented with ^a^ for the Kruskal–Wallis test. ACHD, adult congenital heart disease; CM, cardiomyopathy; HD, heart disease; MACE, major adverse cardiac event; PH, pulmonary hypertension; VHD, valvular heart disease

### Obstetric complications

The obstetric complications in women with (five groups) and without HD are shown in Table 3 and Fig. 3b. The total obstetric complication rate in mothers with HD was higher compared with that in mothers without HD (4.7% vs. 54.8%; *P* = 0.002), and this result was predominantly due to the high prevalence of placental abruption, known or suspected fetal abnormality, and postpartum hemorrhage. However, the women with HD had significantly lower incidence rate of laceration than women without HD (2.8% vs. 8.9%; *P* = 0.001), which was consistent with the low vaginal delivery type in the former group (35.7% vs. 60.7%; *P* <  0.0001). These results may be because laceration is more common in vaginal or assisted delivery among patients without HD than among patients with HD [15].

**Table 3:** Obstetric Complications and NACE outcomes of Women with and without HD in Southwestern China

	HD (*n* = 508)	No HD (*n* = 44,559)	*P* value	VHD (*n* = 66)	CM (*n* = 84)	ACHD (*n* = 207)	PH (*n* = 7)	Other (*n* = 144)	*P* value
Obstetric Complications (%)	64.7	54.8	0.002	57.6	58.8	72.2	50.0	60.3	0.282
Abruptio placenta (%)	2.4	0.5	< 0.0001	3.0	0.0	2.8	0.0	2.9	0.899
Adherent placenta (%)	4.8	3.4	0.213	9.1	2.9	4.6	16.7	2.9	0.289
Breech delivery (%)	5.2	4.0	0.321	0.0	8.8	7.4	0.0	2.9	0.329
DIC (%)	0.8	0.1	0.011	0.0	2.9	0.0	0.0	1.5	0.314
Early or threatened labor (%)	33.7	36.8	0.318	33.3	29.4	30.6	33.3	41.2	0.657
Known or suspected fetal abnormality (%)	10.8	0.6	< 0.0001	6.1	5.9	21.3	0.0	0.0	< 0.0001
Laceration (%)	2.8	8.9	0.001	3.1	0.0	2.8	0.0	4.4	0.804
Long labor (%)	0.0	0.7	0.332	0.0	0.0	0.0	0.0	0.0	.
Placenta insufficiency (%)	0.0	0.1	1.000	0.0	0.0	0.0	0.0	0.0	.
Placenta previa (%)	5.2	3.2	0.076	0.0	8.8	6.5	16.7	2.9	0.167
Precipitate labor (%)	0.4	1.1	0.444	0.0	0.0	0.9	0.0	0.0	1.000
Premature rupture of membranes (%)	13.7	17.0	0.158	18.2	11.8	11.1	16.7	16.2	0.698
Polyhydramnios (%)	1.2	1.0	0.934	0.0	0.0	1.9	0.0	1.5	1.000
Postpartum hemorrhage (%)	6.0	2.0	< 0.0001	3.0	14.7	5.6	0.0	4.4	0.312
Postpartum infection (%)	0.0	0.1	1.000	0.0	0.0	0.0	0.0	0.0	.
NACE (%)	23.3	11.3	< 0.0001	18.2	29.4	23.1	33.3	22.1	0.819
Fetal death (%)	2.8	0.5	< 0.0001	3.0	2.9	2.8	0.0	2.9	1.000
In-hospital death (%)	0.0	0.0	1.000	0.0	0.0	0.0	0.0	0.0	.
Prematurity (%)	12.7	7.1	< 0.0001	9.1	17.6	12.0	33.3	12.3	0.443
Intrauterine growth restriction (%)	3.2	1.3	0.014	6.1	0.0	5.6	0.0	8.8	0.489
Respiratory distress syndrome (%)	8.0	0.3	< 0.0001	9.1	14.7	7.4	0.0	5.9	0.571
Intracranial cerebral events (%)	0.0	0.0	1.000	0.0	0.0	0.0	0.0	0.0	.
Non-NACE variables (%)	4.4	4.5	0.956	3.0	5.9	5.6	16.7	1.5	0.283
Infant with low birth weight (%)	2.0	0.6	0.015	0.0	0.0	2.8	16.7	1.5	0.191
Fetal macrosomia (%)	2.4	3.9	0.228	3.0	5.9	2.8	0.0	0.0	0.330

Data are shown as (%) except as noted. *P* values were based on the Pearson chi-squared test or the Fisher exact test. ACHD, adult congenital heart disease; CM, cardiomyopathy; DIC, disseminated intravascular coagulation; HD, heart disease; NACE, neonatal adverse clinical events; PH, pulmonary hypertension; VHD, valvular heart disease

### Neonatal clinical events

The neonatal outcomes in the offspring of women with and without HD are listed in Table 3 and Fig. 3c. NACEs were more common in the offspring of women with HD (23.3% vs. 11.3%; *P* <  0.0001) compared with those in the offspring of women without HD. Patients with PH and cardiomyopathy experienced high percentages of NACE. This result was primarily due to the increased rates of fetal death, prematurity, and respiratory distress syndrome. Among the patients with HD, the PH group experienced the highest incident rate of prematurity, whereas the VHD group commonly had respiratory distress syndrome. In the apropos of non-NACE variables, no significant difference was observed between women with and without HD (4.4% vs. 4.5%; *P* = 0.956). The infants with low birth weight (weight < 2500 g) but no fetal macrosomia (weight > 4000 g) in mothers with HD were significantly higher compared with those in mothers without HD (2.0% vs. 0.6%; *P* = 0.015). The proportion of fetal macrosomia in the HD group (2.4%) showed a lower trends than that in the non-HD group (3.9%) but no significant difference (*P* = 0.228), while the incidence of diabetes mellitus in the HD group (7.9%) was lower than that in the non-HD group (14.8%) (*P* <  0.0001). This result may be influenced by the diabetes mellitus of mothers because previous studies have reported their association [16]. The higher rate of infants with low birth weight (P = 0.015) was consistent with the higher rate of prematurity (*P* <  0.0001) in the HD group (2.0% and 12.7%, respectively) compared with the non-HD group (0.6% and 7.1%, respectively).

### HF

Of the 508 patients with HD, 73 (14.4%) developed HF during pregnancy or after delivery. Incident HF was highest in women with PH (42.9%) during pregnancy or after delivery compared with the four other types from Table 2. The baseline characteristics of patients with and without HF are shown in Table 4. The BMI (*P* = 0.003), heart rate (*P* <  0.0001), BNP (P <  0.0001), total hospital charges (*P* = 0.001), and percentage of cardiac medication use (*P* <  0.0001) of the HF group were significantly higher than those of the non-HF group. For the echocardiographic outcomes, the patients with HF had a lower fractional shortening (*P* <  0.0001), lower ejection fraction (*P* <  0.0001), and higher left ventricular end-diastolic diameter (*P* = 0.002) than the patients without HF.

**Table 4:** Baseline Characteristics of Primigravid Women with Cardiac Disease with and without HF in Southwestern China

	HF (*n* = 73)	No HF (*n* = 435)	*P* value
Age (years)	30.5 ± 5.5	30.5 ± 5.3	0.907^a^
Age group (years)			
18–25	10 (13.7)	71 (16.3)	0.748
26–35	51 (69.9)	283 (65.1)	
> 35	12 (16.4)	81 (18.6)	
Various etiologies classification			
VHD	17 (23.3)	49 (11.3)	< 0.0001
CM	18 (24.7)	66 (15.2)	
ACHD	28 (38.4)	179 (41.1)	
PH	3 (4.1)	5 (0.9)	
Other	7 (9.6)	137 (31.5)	
Smoking (%)	1.9	1.8	1.000
Drinking (%)	3.8	0.9	0.159
BMI (kg/m^2^)	28.8 ± 5.7	25.9 ± 3.2	0.003^a^
Obesity (%)	11.8	1.1	0.065
Hypertension known before pregnancy or present in the first 20 weeks	0 (0.0)	3 (0.7)	1.000
Hypertension arising de novo at or after 20 weeks	13 (17.8)	16 (3.7)	< 0.0001
Diabetes mellitus	10 (13.7)	30 (6.9)	0.046
NYHA functional class			
I–II	51 (69.9)	433 (99.5)	< 0.0001
III–IV	22 (30.1)	2 (0.5)	
Heart rate (bpm)	96.5 ± 19.2	85.7 ± 15.4	< 0.0001^b^
SBP (mmHg)	117.7 ± 17.5	118.5 ± 35.3	0.869^a^
DBP (mmHg)	75.7 ± 13.4	76.6 ± 25.4	0.980^a^
Echocardiographic outcomes			
Ejection fraction (%)	57.5 ± 11.1	66.0 ± 6.1	< 0.0001^b^
Fractional shortening (%)	31.0 ± 6.9	36.1 ± 5.7	< 0.0001^a^
Left ventricular end-diastolic diameter (mm)	50.6 ± 10.3	45.3 ± 5.5	0.002^b^
LDL (mmol/l)	2.4 ± 0.2	2.4 ± 0.7	0.722^b^
TG (mmol/l)	2.2 ± 1.3	2.2 ± 1.9	0.914^a^
TC (mmol/l)	4.8 ± 0.6	4.4 ± 1.4	0.450^a^
BNP (pg/ml)	3262.3 ± 7390.7	282.0 ± 624.3	< 0.0001^b^
Total hospital charge (RMB)	9299 (6135, 13,485)	5855 (3701, 9131)	0.001^b^
Cardiac medication (all) (%)	47.1	14.3	< 0.0001
Diuretics (%)	36.8	6.0	< 0.0001
β-blocker (%)	17.6	3.7	< 0.0001
Anticoagulation (%)	11.9	3.7	0.006
Digitalis (%)	8.8	1.0	0.001
Calcium channel blocker (%)	13.2	5.3	0.038

Data are shown as mean ± SD or median(IQR) for continuous outcomes and *n* (%) for categorical outcomes. P values were based on one-way ANOVA or the Kruskal–Wallis test for continuous outcomes and the Pearson chi-squared test or the Fisher exact test for categorical outcomes. The test utilization in continuous variables was presented with ^a^ for ANOVA and ^b^ for the Kruskal–Wallis test. ACHD, adult congenital heart disease; BMI, body mass index; BNP, brain natriuretic peptide; CM, cardiomyopathy; DBP, diastolic blood pressure; HF, heart failure; LDL, low density lipoprotein; NYHA, New York Heart Association; PH, pulmonary hypertension; SBP, systolic blood pressure; TC, total cholesterol; TG, total triglyceride; VHD, valvular heart disease

### Predictors of HF

The results of the univariable and multivariable logistic regression analyses are shown in Table 5. Age > 35 years, drinking, obesity, hypertension disorders of pregnancy, diabetes mellitus, NYHA classification ≥3, heart rate ≥ 100 bpm, echocardiographic parameters, and cardiac medication use were included as factors for analyzing the association with HF in the univariable model. Hypertension (arising de novo at or after 20 weeks) was an associated factor of HF in the univariate analysis (OR = 5.7, 95% confidence interval [CI] = 2.6–12.4; *P* <  0.0001) but was not significantly associated with HF in the multivariate analysis. A similar result was found in cardiac medication use, which was significantly associated with HF in the univariate analysis (OR = 5.4, 95% CI = 3.0–9.4; P <  0.0001) but not in the multivariable analysis. In a multivariable model, the significant independent parameters associated with HF were NYHA class ≥3 (OR = 15.9, 95% CI = 2.5–99.7; *P* = 0.003), heart rate ≥ 100 bpm (OR = 3.8, 95% CI = 1.1–13.5; *P* = 0.036), ejection fraction ≤60% (OR = 6.4, 95% CI = 2.0–21.0; *P* = 0.002), and left ventricular end-diastolic diameter ≥ 50 mm (OR = 3.4, 95% CI = 1.1–11.2; *P* = 0.041).

**Table 5:** Potential predictors of HF

Univariable	OR (95% CI)	*P* value
Age > 35 years	.	0.655
Drinking	.	0.105
Obesity	.	0.064
Hypertension known before pregnancy or present in the first 20 weeks	.	0.477
Hypertension arising de novo at or after 20 weeks	5.7 (2.6, 12.4)	< 0.0001
Diabetes mellitus	2.1 (1.0, 4.6)	0.05
NYHA functional class ≥ III	93.4 (21.3, 408.8)	< 0.0001
Heart rate ≥ 100 bpm	4.1 (2.1, 8.0)	< 0.0001
Ejection fraction ≤60%	9.2 (4.0, 20.7)	< 0.0001
Fractional shortening ≤32%	6.6 (3.0, 14.7)	< 0.0001
Left ventricular end-diastolic diameter ≥ 50 mm	6.1 (3.0,13.0)	< 0.0001
Cardiac medication use	5.4 (3.0, 9.4)	< 0.0001
Multivariable		
Hypertension arising de novo at or after 20 weeks	.	0.788
Diabetes mellitus	.	0.627
NYHA functional class ≥ III	15.9 (2.5, 99.7)	0.003
Heart rate ≥ 100 bpm	3.8 (1.1, 13.5)	0.036
Ejection fraction ≤60%	6.4 (2.0, 21.0)	0.002
Left ventricular end-diastolic diameter ≥ 50 mm	3.4 (1.1, 11.2)	0.041
Cardiac medication use	.	0.127

HF, heart failure; NYHA, New York Heart Association. For both models, an odds ratio > 1 indicated that one category had higher risk of having HF than the reference category, and odds ratio < 1 indicated that one category had lower risk of having HF than the reference category. “.” represented insignificant variables that were not included in the model

## Discussion

The present study showed the current prevalence of HD (including VHD, cardiomyopathy, ACHD, PH, and other cardiac conditions) and its maternal, obstetric, and neonatal complications in primigravid women in Southwestern China. Only the women’s earliest singleton deliveries during the study period were analyzed to avoid the possible confounding effect of multiple birth or multiparity. The patients with HD had higher rates of MACEs compared with the patients without HD, in which the patients with PH experienced the highest percentage. Obstetric complications were more common in women with HD than in women without HD. The rates of NACEs, including prematurity and respiratory distress syndrome, in mothers with HD (predominantly those with PH) were significantly higher than those in mothers without HD. Developing HF was highest in the PH group during pregnancy or after delivery compared with the four other types. The NYHA class ≥3, heart rate ≥ 100 bpm, ejection fraction ≤60%, and left ventricular end-diastolic diameter ≥ 50 mm were independently associated with a high risk of incident HF.

### Patient characteristics and outcomes

A prior study of > 2.2 million admissions for delivery have reported the prevalence of HD and its in-hospital outcome in the New York State during a 15-year period [17]. The differences in the composition of HD and its characteristics were detected. For the total HD distribution, VHD (40%) and ACHD (35%) predominated in the New York State. However, ACHD (41%) and other cardiac conditions (28%) were more common than the rest forms, and only 1% proportion of PH remained in Southwestern China. Besides, the present study showed that diabetes mellitus was more common in mothers without HD than in mothers with HD, which was conversely different from the current state in New York. Significantly advanced age and higher percentage of Han nationality and hypertension (arising de novo at or after 20 weeks) have been identified as risk factors for gestational diabetes mellitus development in a previous study of Chinese population [18]. However, these factors were detected in women without HD in the present analysis. We considered the findings above responsible for the difference in diabetes mellitus. As a result, fetal macrosomia was affected and consistent with the trend of diabetes mellitus. Furthermore, the study from the New York State has shown that neonatal complications tend to follow a pattern similar to the maternal and obstetric outcomes. These outcomes are found to be highest in the offspring of patients with cardiomyopathy and PH and low in the offspring of patients with ACHD and VHD [17]. By contrast, the present study showed that patients with PH had the highest rates of MACEs and NACEs. Among the rest types of HD, the cardiomyopathy group was observed with relatively higher incidence of neonatal outcomes. A decline in the maternal cardiac output in women with HD during pregnancy and abnormal umbilical artery Doppler flows, which independently predict neonatal complications, have been previously shown to result in placental ischemia particularly in women with cardiomyopathy [19, 20]. For patients with PH, the causes of poor maternal and neonatal outcomes are varied and include the risk of death from right HF and stroke from intracardiac shunting [21]. The hemodynamic (an increase in blood volume, red cell mass, left ventricular mass, and cardiac output), hemostatic (an increase in coagulation factors and fibrinogen), and hormonal (an increase in levels of progesterone and estrogen with vasodilatory effects) changes can put a considerable strain on the right ventricle and lead to right ventricular failure in pregnant women with PH [8, 22–24]. Furthermore, the high peri−/postpartum risk due to hemodynamic stress and bleeding complications can lead to right HF [25, 26]. In this scenario, premature birth and growth retardation are reported in successfully delivered children [27]. The same result was also detected in our study. The percentage of prematurity and the low birth weight of infants in mothers with HD were significantly higher than those in mothers without HD, and PH predominated evidently. Respiratory issues, asthma, developmental delays, intestinal problems, infections, hearing loss, and retinopathy in newborns and children may be presented as a result [28].

### HF

In the analysis, the patients in the PH group were more likely to develop HF than those in the non-PH group. PH has been recognized as a high-risk condition and predictor for cardiovascular events in previous studies, [26, 29] which has been also confirmed recently in an large international observational registry [9]. The inability of patients with PH to tolerate any change discussed before pregnancy is attributed to a high risk of HF. Moreover, the current guidelines clearly recommend the avoidance of pregnancy in women with PH and termination when pregnancy occurs [30].

### Predictors of HF

Of the factors that remained significant in multivariable regression, the strongest were poor functional class and reduced ejection fraction. Cardiac output increased by 30–50% during pregnancy, reaching a peak in the second trimester that was maintained until term [31]. In patients with HD, these changes contributed to a persistent reduction in systolic and diastolic cardiac functions. Consistent with our finding, significantly increased cardiac output, stroke volume, left ventricular end-diastolic diameter, left ventricular mass, and *E/E′* ratio and decreased ejection fraction and fractional shortening are observed during pregnancy [32, 33]. These results suggested a reduced cardiac potential for adaptation to the normal requisites of pregnancy in women with HD, facilitating the progression of incident HF.

Increased heart rate is a frequent symptom of HF and considered a poor prognostic indicator [34]. Our present study identified heart rate ≥ 100 bpm as a risk factor for HF, which was consistent with increased blood volume and cardiac output and decreased systemic vascular resistance and blood pressure during pregnancy [22, 23, 35, 36].

BNP is established as a biomarker of HF [37]. A previous study has shown that the BNP levels are associated with cardiovascular events during pregnancy [38]. In the present study, the BNP levels in women with HF were significantly higher than those in women without HF. As for neonatal complications, Bhatla et al. have shown that more fetal complications and preterm and low-birth weight neonates are reported among patients in the NYHA class III/IV than those among patients in the NYHA class I/II [39]. MACEs are independently associated with a high risk of incident NACE among women with HD [17]. These findings were confirmed in our analysis.

### Clinical significance

The present study depicted the current prevalence and the in-hospital complications of primigravid women with HD during the past 9-year period in Southwestern China. Our observations highlighted the risk factors of having HF and neonatal complications in patients with HD especially those with PH. These results indicated the regular monitoring of potential predictors to reduce maternal and neonatal complications. The strengths of our study lied in the differences in disease composition and its characteristics of primigravid women with HD between the two centers of eastern and western countries and the prospective risk assessment for incident HF to attach importance on early diagnosis and management of HD in developing countries.

### Study limitations

Several limitations should be considered in the present study. First, some potentially useful data were unavailable (e.g., individuals <10th percentile for gestational age, socioeconomic level, and education), and some detected data were missing due to the retrospective design and some limitations of the database. Second, the outcomes (e.g., echocardiography) that may be subjective were dependent on the assessment of cardiologist and/or technologist because the data varied from seven medical centers with different levels of diagnosis and treatment. Finally, these findings cannot be used to establish a conclusive cause-and-effect relationship between potential predictors and incident HF because this study was a cross-sectional study since women with HF prior to pregnancy were not excluded. Hence, values reported in the present study may be overestimated if these women are at greater risk of developing hypertensive disorders of pregnancy.

## Conclusion

In the present study of 45,067 primigravid women in Southwestern China during a 9-year period, women with HD (including VHD, cardiomyopathy, ACHD, PH, and other cardiac conditions [such as primary arrythmia, coronary HD, anemic HD, and hyperthyroid HD]) experienced higher rates of MACE and obstetric complications compared with women without HD. The rates of NACEs, including prematurity and respiratory distress syndrome, in the offspring of mothers with HD were also significantly higher compared with those in the offspring of mothers without HD. The incident MACEs and NACEs in women with PH were highest compared with those in women with other types of HD. NYHA class ≥3, heart rate ≥ 100 bpm, ejection fraction ≤60%, and left ventricular end-diastolic diameter ≥ 50 mm were independently associated with a high risk of incident HF. Given the higher rate of these in-hospital complications and predictors of having HF, women with cardiac disease should undergo evaluation of their disease subtype and cardiac function earlier and should be treated in centers with expertise on a multidisciplinary approach.

## Supplementary information

**Additional file 1: Table S1.** Data Extraction. ICD-10 Codes Used for Variables Definition.

## Data Availability

The data that support the findings of this study are available from the Yiducloud (Chongqing) Technology Co., Ltd. and the cooperative Medical Data Science Academy of Chongqing Medical University. These data, which were used under license for the current study, are restricted and not publicly available. The datasets used and/or analyzed during the current study are available from the corresponding author on reasonable request.

## Abbreviations

ACHD: Adult congenital heart disease
BNP: Brain natriuretic peptide
HD: Heart disease
HF: Heart failure
MACE: Maternal major adverse cardiac events
NACE: Neonatal adverse clinical events
NYHA: New York Heart Association
PH: Pulmonary hypertension
VHD: Valvular heart disease
