# Supplementary material for: Real-world in-hospital outcomes and potential predictors of heart failure in primigravid women with heart disease in Southwestern China
Source: BMC Pregnancy Childbirth. 2020 Jun 23;20:372. doi: 10.1186/s12884-020-03058-9 (PMC7310540; doi:10.1186/s12884-020-03058-9)
Supplement: Supplementary file 1 — Additional file 1: Table S1. Data Extraction. ICD-10 Codes Used for Variables Definition. [file 12884_2020_3058_MOESM1_ESM.docx]

Table S1. Data Extraction. ICD-10 Codes Used for Variables Definition.

1 Heart Disease

Cardiomyopathy: I42, I43

Valvular Heart Disease (VHD): I34, I35, I36, I37, I38, I05, I06, I07, I08, I09

Congenital Heart Disease (CHD): Q24, Q25, Q26, O99.810

Pulmonary Hypertension (PH): I27.0, I27.2, O99.404

Other cardiac conditions (primary arrythmia, coronary HD, anemic HD, and hyperthyroid HD): I44, I45, I46, I47, I48, I49, I25.1, I25.2, D64.910+, E05.905+

2 Patient characteristics

Smoking: Z72.0

Drinking: Z72.1

Obesity: E65, E66

Hypertensive Disorder of Pregnancy: O10, O11, O12, O13, O14, O15

Diabetes: E10, O24

Delivery type: Vaginal: O80, O81, O83; Cesarean: O66.401, O82

3 Outcomes

3.1 Major adverse cardiac event (MACE)

Arrythmia: I4, I45, I46, I47, I48, I49, O99.424

Shock: I57

Cerebral Events: I61, I62, I63, I64, I65, I66, I67, I68, I69

Heart Failure (HF): I50, O99.425

Respiratory Failure: J96

Pulmonary Embolism: I26, O88.202

Dissection of any artery: I67, I71

3.2 Non-MACE outcomes

Acute renal failure: N17

3.3 Obstetric Complications

Abruptio placenta: O45

Adherent placenta: O72.002, O73.001

Breech delivery: O80.1, O83.1

Disseminated intravascular coagulation (DIC): D65, O46.001, O72.301

Early or threatened labor: O60

Known or suspected fetal abnormality: O35

Laceration: O70, O71.3, O71.4

Long labor: O63

Placenta insufficiency: O43

Placenta previa: O44

Precipitate labor: O62.3

Premature rupture of membranes: O42

Polyhydramnios: O40

Postpartum hemorrhage: O72

Postpartum infection: O86

3.4 NACE

Fetal death: P21, P95.x01

Prematurity: P07.3

Intrauterine growth restriction: P05.901

Respiratory distress syndrome: P22

Intracranial cerebral events: P10, P11, P12, P52, P39.802

3.5 Non-NACE variables

Infant with low birth weight: P05.001, P07.0, P07.1

Fetal macrosomia: P08
